# Development of the Electronic Social Network Assessment Program Using the Center for eHealth and Wellbeing Research Roadmap

**DOI:** 10.2196/humanfactors.7845

**Published:** 2017-08-30

**Authors:** Maija Reblin, Yelena P Wu, Justin Pok, Lauren Kane, Howard Colman, Adam L Cohen, Eduardo Mendivil, Echo L Warner, Miriah Meyer, James Agutter

**Affiliations:** ^1^ Department of Health Outcomes & Behavior Moffitt Cancer Center Tampa, FL United States; ^2^ University of Utah Salt Lake City, UT United States; ^3^ Huntsman Cancer Institute Salt Lake City, UT United States

**Keywords:** intervention development, user-centered design, oncology, caregiver

## Abstract

**Background:**

The number of Web-based psychological and behavioral interventions is growing. Beyond their theoretical underpinnings, a key factor to the success of these interventions is how they are designed and developed to ensure usability over a new method of delivery. Our team has adapted ecomapping, a tool for visualizing family caregiver social network resources, for the Web. Here, we describe how we designed and developed the electronic Social Network Assessment Program (eSNAP) Web-based tool using a framework of the Center for eHealth and Wellbeing Research (CeHRes) Roadmap for Web-based intervention development. The CeHRes Roadmap is still new in terms of tool development and we showcase an example of its application.

**Objective:**

The aim of our study was to provide an example of the application of the Web-based intervention development process using the CeHRes Roadmap for other research teams to follow. In doing so, we are also sharing our pilot work to enhance eSNAP’s acceptance and usability for users and the feasibility of its implementation.

**Methods:**

We describe the development of the eSNAP app to support family caregivers of neuro-oncology patients. This development is based on the 5 iterative stages of the CeHRes Roadmap: contextual inquiry, value specification, design, operationalization, and summative evaluation. Research activities to support eSNAP development prior to implementation included literature review, focus groups, and iterative rounds of interviews.

**Results:**

Key lessons learned in developing the eSNAP app broadly fell under a theme of translating theoretical needs and ideas to the real world. This included how to prioritize needs to be addressed at one time, how the modality of delivery may change design requirements, and how to develop a tool to fit within the context it will be used.

**Conclusions:**

Using the CeHRes Roadmap to develop Web-based interventions such as eSNAP helps to address potential issues by outlining important intervention development milestones. In addition, by encouraging inclusion of users and other stakeholders in the process, Web-based intervention developers using the Roadmap can identify what will work in the real world and increase feasibility and effectiveness.

## Introduction

Web-based health interventions can increase knowledge, adherence to treatment regiments, and patient empowerment [1,2] by addressing access, privacy, and scalability barriers found in traditional, in-person interventions such as education, therapy, or support group sessions. However, not all Web-based interventions are successful; some fail to produce an effect, while others simply fail to become implemented or integrated into practice [3]. While the scientific quality of the intervention is essential to success, ensuring that a Web-based intervention is designed appropriately is also a key factor [4].

**Figure 1:**
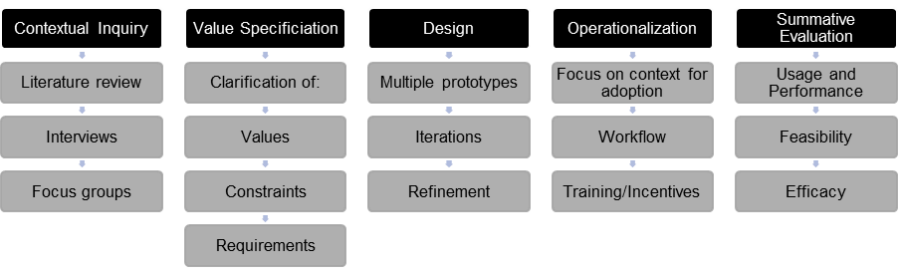
Stages of the CeHRes Roadmap with research tasks.

Here, we describe steps outlined in the Center for eHealth and Wellbeing Research (CeHRes) Roadmap for Web-based intervention development [5] and how we applied the steps in our work. The CeHRes Roadmap was established based on evaluation of prior frameworks, empirical evidence, and expert input [5]. The roadmap takes an iterative approach through 5 phases of development: contextual inquiry, value specification, design, operationalization, and summative evaluation (Figure 1). As this is a new model of tool development, exemplars of its application are needed.

Our goal was to describe how our interdisciplinary team, consisting of behavioral scientists, designers, and computer programmers, applied the CeHRes Roadmap to partner with social workers and family caregivers and develop the electronic Social Network Assessment Program (eSNAP). eSNAP is a Web-based social network assessment tool grounded in theory, designed to support family caregivers of patients with primary brain tumor. By sharing our pilot work in this process, we hope other research teams will benefit from the example and our lessons learned. All research activities were conducted under institutional review board (IRB) approval.

## Methods

The CeHRes Roadmap provided the framework for our intervention development. Because the roadmap is iterative and exploratory, we conducted several small studies with various methodologies across the 5 steps. The goal of the first step of the roadmap, contextual inquiry, is to gain an understanding of prospective users, the problem they face, and how one might solve that problem. In order to verify the findings of our literature review, we conducted a focus group with providers and interviews with family caregivers, both of whom we considered important stakeholders. The second step, value specification, is meant to clarify values, constraints, and requirements—what is important to include in the tool and how it should work. To clarify these, we conducted another round of purpose-driven interviews with family caregivers and providers. In later iterations of these interviews, we were also able to identify an information architecture, which helped us moving into the third step, design. Digital prototypes were developed and presented to family caregivers to give feedback on design and flow of the experience. Iterations were tested as new features were added until caregivers were unable to suggest features to improve the tool. At this point, we moved to the fourth step, operationalization, which involves introducing the technology into practice. We are currently conducting a feasibility trial to gather information about implementation of our tool in the real world and collecting preliminary outcome data, to address the fifth step, summative evaluation.

## Results

### Contextual Inquiry

Contextual Inquiry involves gaining an understanding of prospective users and their context. This includes defining the problem, gathering input about how to solve the problem, and gaining an understanding of relevant environmental factors. Our team used a variety of approaches to address the goals within this stage, including a review of the literature, interviews, and focus groups.

#### Literature Review

Family caregivers relieve demands on the formal healthcare system by caring for patients at home. Often, the family member who spends the most time caregiving is a spouse, but can also be an adult child, parent, or other individual. [6] The majority of cancer caregivers are women and on average they are in their mid-50s. [7] While some report benefiting from providing care (eg, learning new skills, strengthening relationships) [8], there is evidence that informal caregiving can be burdensome [9-11] and stress associated with caregiving can adversely affect quality of life, psychological and physical health [12-14], and patient outcomes [15,16]. Caregivers of patients with primary brain tumor are at particular risk for high burden, given the low survival rate, rapid status changes, and cognitive and emotional impact of the disease [17,18]. In addition, this population often receives little attention in research.

The caregiving stress process model [19] and research evidence [20,21] suggest that a potential solution to reduce caregiver burden is the provision of adequate social support from family caregivers’ existing networks of friends, family, and others (eg, information or help problem solving), emotional support (eg, “being there” or validation), and instrumental support (eg, assistance with household tasks). Caregivers who report adequate support have better health and quality of life [22,23]. Thus, the specific problem we chose to address was that, despite the value of support, caregivers—especially caregivers of patients with primary brain tumor—often cut themselves off from their social networks or fail to take advantage of available support to focus on providing care independently [23,24].

To address this issue, healthcare providers have been urged by the Institute of Medicine to assess caregiver social connections [25] and to facilitate use of social resources to reduce burden [26]. Yet, the systematic assessment of caregivers’ social resources is not yet integrated into routine clinical practice. A primary barrier is a lack of efficient and user-friendly clinical tools to collect and process this information [11]. Thus, caregivers’ social network resources (or lack thereof) are typically invisible to providers [12]. Further, if providers do not engage in discussions with caregivers about social resources, critical information is missed that may impact patient care decisions.

Prior work has outlined several other approaches to increase support. For example, some studies focused on increasing support between patient and caregiver [27]; however, this approach did not seem feasible for a primary brain tumor population since patients may be unable to provide support. Other teams focused on providing Web-based information or support groups, which can provide benefit [28,29] but do not address or leverage the existing social network. Finally, some teams developed tools focused on helping caregivers identify and problem-solve their needs [30]. This seemed like an effective, practical approach for our population; however, a continued barrier is identifying resources within caregivers’ existing networks that could provide reliable, valued assistance [31].

**Figure 2:**
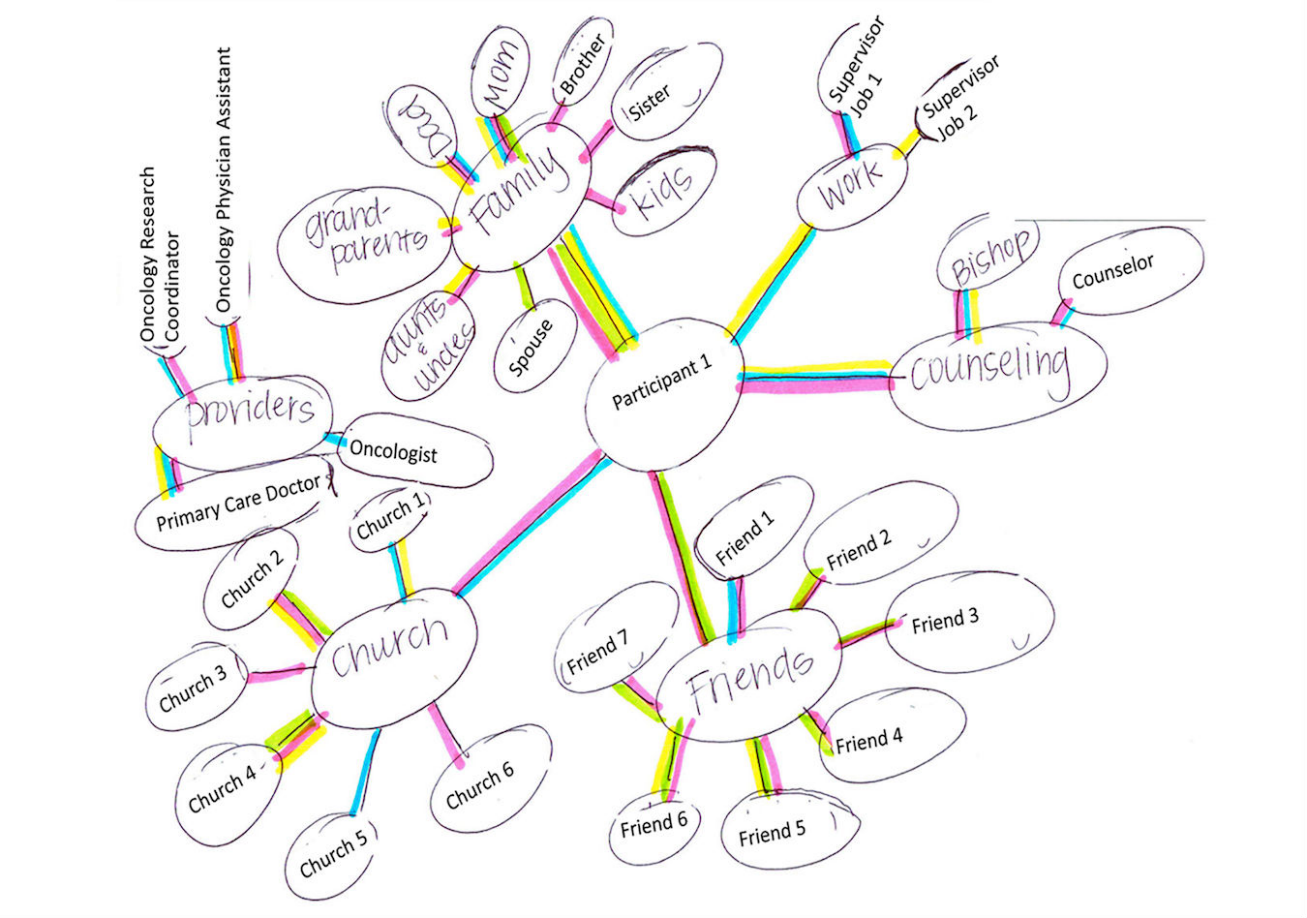
Example ecomap.

Ecomapping is a social work tool for visualizing an existing social network (Figure 2). It organizes and depicts information about that network’s size, strength, quality, and function, and can highlight barriers to support, such as social isolation or failure to take advantage of existing support [32]. Visual representation off-loads the cognitive burdens of building and storing mental maps of relationships and allows the perceptual system to quickly search for relationships of interest [33]. Social network visualization can prime or create implicit associations to the availability of these resources.

**Figure 3:**
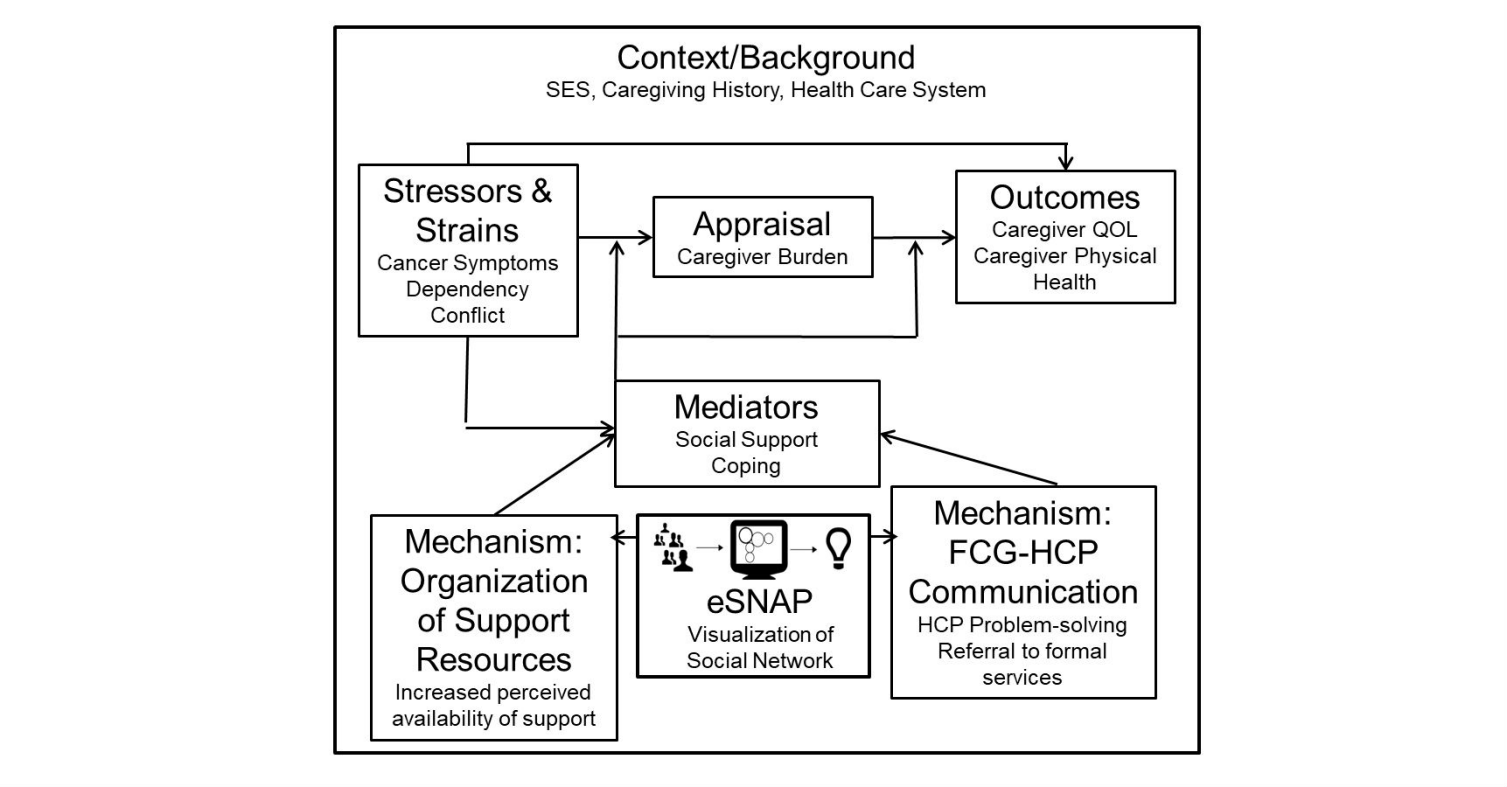
Conceptual model.

Specific benefits of using ecomaps have included caregivers identifying unrealized social resources and facilitating provider-caregiver communication and rapport [34-36]. Through visualization, both caregivers and providers can quickly understand caregiver needs and existing resources and providers can be better prepared to help caregivers more effectively and efficiently problem solve the use of existing resources or refer to formal support services. We elected to modify this tool and improve it through automation as a means to solve the problem of caregiver social resource use. Our conceptual model, based on the stress process model [19], is shown in Figure 3. Using our tool to create a visualization of a caregiver’s social network is expected to help organize social support resources and facilitate caregiver-provider communication. Through both of these mechanisms, we expect that social support will increase, which will buffer the negative impact of objective stressors and strains on caregiver quality of life and physical health by reducing the appraisal of subjective burden.

To verify this approach and ensure we had considered all the important factors, we conducted pilot interviews and a focus group.

#### Caregiver Interviews

Four spouse caregivers of neuro-oncology patients undergoing treatment at a National Cancer Institute (NCI) designated Comprehensive Cancer Center were interviewed about social support needs and caregiving. Caregivers were also introduced to the concept of ecomapping and asked their opinions about whether they thought visualization would be helpful in changing how they used their support networks. All participants were female with a mean age of 35.5 years (SD 5.5). The mean length of the relationship between caregivers and spouses was 13 years (SD 2.2).

**Table 1:** User needs, tool requirements, and feature specifications of eSNAP.

User need	Tool requirement	Feature specifications
Understand the need for support when caregiving	Informative introduction with explanation of purpose	4-page tailored introduction with form, icons, text, and buttons
Understand types of support	List and explain each type of support separately to avoid information overload	Nest data in separate tabbed containers for each category of support
Write down people that can offer support to the user	Provide forms to create lists for each category of support	Include forms within each tabbed container
Evaluate effectiveness of support	Mark the level of helpfulness for each person entered	Add a position slider to rank 1 of 3 levels of effectiveness. Default to “Somewhat Helpful”
Identify the strengths of users’ network	Rate each category of support based on number and helpfulness of network members	Include a summary page to rate strength of each support category, based on number of people and helpfulness
Ability for user to take the network with them	Include a printable summary page	Format summary data into printer friendly version
Identify areas of network that can use additional support	Identify the types of support that may benefit from additional resources and provide information	Provide a link to resources for each category that has less than 2 stars on the summary page
Find specific supportive resources	Provide lists of resources for each type of support	Include a backend editable database with caregiver resources
Maintain support network changes and additions	Begin with end in mind by building tool on Web-based technologies ensuring accessibility across various platforms and devices	Design a Web app compatible with Mac and Windows OS, with text and elements sized for various landscape screen sizes.

Caregivers reported discussing support resources with neuro-oncology team members but were hesitant to initiate these discussions. When discussions about support resources occurred, they were viewed as insufficient in terms of time and depth. Consistent with findings in other populations [34,35], caregivers verified that social support was very important but often perceived as lacking. Caregivers felt that it was stressful to identify and organize available resources on their own, but felt that having some kind of visualization of their support network would be helpful. Caregivers also indicated that support changed over time and recommended that an exercise to identify and visualize support be done early on and modified as needed. However, one problem identified by caregivers using traditional ecomaps was that the visualizations tended to be messy; it was difficult to expand the Web to include many resources and there often wasn’t a consistent logic to where different resources were placed.

#### Provider Focus Group

A focus group of neuro-oncology clinic members, including 2 physicians, 1 nurse, 1 medical assistant, and 1 social worker was conducted to discuss social support needs of family caregivers and the utility of social support network visualization. The feasibility of implementing research in the clinic was also discussed.

Providers confirmed that creating a visualization was one way to facilitate in-depth support discussions between caregivers and nurses or social workers; some team members already had familiarity with the concept of ecomapping. All team members mentioned that having this information available, at least to some members of the team, would be helpful and visualizations would save time over gathering narratives. Barriers to ecomap use included time, the need to maintain clinic workflow, and potential challenges in addressing issues raised by caregivers.

### Value Specification

After outlining our problem and identifying and verifying a potential solution, we moved to value specification: identifying the most important stakeholder values to be translated into user requirements. User requirements are detailed descriptions of what has been identified by users as important aspects of the tool. Some initial values were identified within the previous interviews, including the desire to refer back to the visualization and change it over time and the need for the tool to be easy to use both independently and in contexts with available medical professionals, while not interrupting clinic workflow. To follow up on these preliminary interactions with caregivers and healthcare providers, we conducted more detailed and purpose-driven interviews with clinic social workers and family caregivers. These helped to define user needs, tool requirements, and feature specifications (Table 1). These interviews also helped shape the language and scope of the tool.

In discussions with oncology social workers and case managers, we found that professionals were concerned with caregiver burnout and wanted this more specifically addressed. Thus, we refined an existing category of “companionship support” to better reflect resources that help promote self-care activities. Similarly, in interviews with caregivers, they mentioned that some of their resources were valued for their ability to share information (eg, sending patient updates) or coordinate others (eg, organizing dinner drop-off). In response, we added a “communication support” category and noticed caregivers were more likely to refer to instrumental support with a more casual term. The decision we made was to change instrumental support to “hands on” support to better fit with the natural terminology.

### Design

Design involves developing prototypes of the technology interface that conform to the user values and specific technical specifications derived from the previous stage. Design can be evaluated at a system level for user-friendliness, content level for tailored, meaningful information, and service level for responsiveness and feasibility for use in the environment.

Our first step was to identify mental models for information architecture or the most logical process to collect and present the information within the tool. We presented caregivers and social workers information processes used in developing the ecomap (grouping people, identifying individuals who can offer support, and identifying types of support) in random order and asked them to order them to reflect their preferred process for data input. We discovered, contrary to how paper-based ecomaps were created, that the most frequent order was to start with the type of support needed, followed by listing individuals who could provide that type of support; few people felt the need to group people.

**Figure 4:**
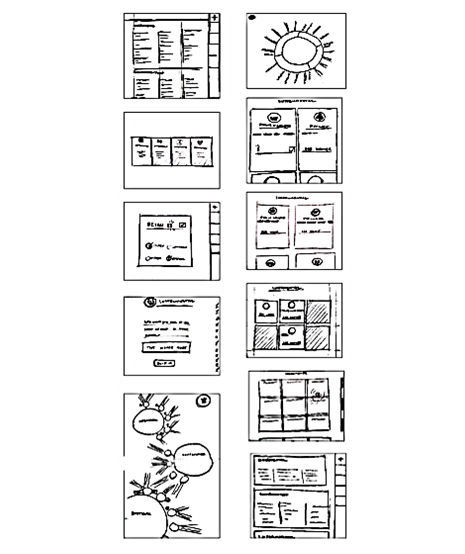
Preliminary sketch concepts.

**Figure 5:**
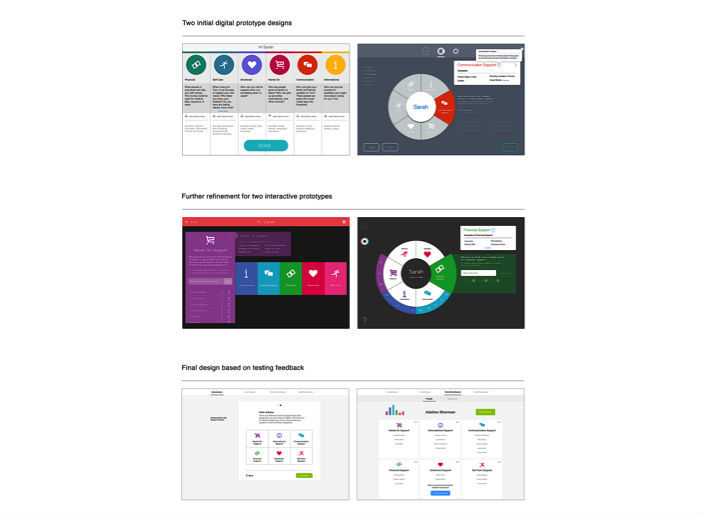
Iterations of prototype design.

Based on the previously identified constraints, requirements, and information architecture, the team produced 4 preliminary sketch concepts (Figure 4) that explored options for the design. Each sketch was internally evaluated and 2 moved on to development as digital prototypes for caregiver evaluation sketches (Figure 5). One digital prototype version took visual form as a set of lists for each category of support to appropriately match caregiver’s mental models. The other version consisted of a pie chart, which dynamically changed with the input of more information to increase user engagement.

The digital prototypes were presented and tested with caregivers who were asked to give feedback on the design and flow of the experience. The amount of time caregivers tested the prototypes varied by stage. Early on when deciding on an information architecture and general design concept, sessions were relatively short (approximately 10 minutes), but later some participants spent up to 30 minutes with the Web-based prototype. We assessed effectiveness (successful completion of tasks) and efficiency (time to learn and carry out an action) and we collected comments through open-ended questions. The design was iteratively updated based on feedback. Of the 10 caregivers that were interviewed, 9 (90%, 9/10) provided demographic information, 77% were female (7/9), and the mean age was 52.3 (SD 11.8) years. All were non-Hispanic white, half were employed full-time, and 66% (6/9) had at least some college education. Most caregivers were spouses of patients, but 2 (20%, 2/10) were adult children and 1 (10%, 1/10) was a parent of the patient.

Both versions were shared initially with caregivers; however, there was a clear and unanimous preference for the list version after 3 interviews. As a result, we chose to pursue the list version of the design for further development. Users also told us they wanted some element to show when they were finished using the app. In response, we added a summary and evaluation page where we incorporated the wheel element; this version was evaluated much more positively.

Consistent suggestions gathered in the open-ended feedback included adding resources beyond the user’s social network. We decided to add a database of supportive resources into the app. Caregivers who tested this feature were able to bookmark contact information for more formal support resources. After testing the new version that included the additional external resources, caregivers were unable to suggest additional features to improve it.

A Web-based app was selected over a native mobile app designed for a particular operating system to allow for flexibility and more accessibility. The design prototype of the app built in InVision was provided to the Web developer. The app was built closely following the specifications provided. This Web-based app was built using Hypertext Markup Language (HTML), Cascading Style Sheets (CSS), and Javascript, and it uses the browser session storage property to store data temporarily, which is removed once the app’s browser tab is closed. The app can be used on the most popular browsers; however, it is recommended to be used in the Chrome browser for the best user experience.

### Operationalization

Operationalization involves the introduction and use of the technology in practice. This includes factors such as training, incentives, and a plan for adoption. While the ultimate goal is for eSNAP to be a standard clinical tool available for use in clinics and connected to the electronic medical record, the current goal is to implement it as a research tool so that we can test its effect on caregiver support, burden, and well-being. To this end, the introduction of eSNAP is somewhat facilitated. Namely, research studies are common in the cancer center environment and clinicians are incentivized to promote participation. Families who receive care at these institutions are also used to being approached to participate in research. As part of the informed consent process, researchers are able to explain the tool being tested, its purpose, and benefits to participation. Moreover, funded research often allows for small participant compensation. Given these incentives, as well as the ability for people to learn about and habituate to the program, electronic health (eHealth) tools that are developed and implemented through research have some advantages, though an eye towards broader implementation and dissemination to the community is also important.

To further encourage use of eSNAP in the cancer center, we engaged clinical stakeholders, including social workers, to ensure buy-in and prevent gatekeeping, and are documenting issues encountered by the research staff in using eSNAP within a clinical setting (ie, problems with connectivity or interruptions that occur as caregivers use the tool). These notes can help determine the appropriate time and place to approach future caregivers without disrupting clinic flow, one of the values of the provider team. This information can also guide the next steps of eSNAP development as we prepare for a larger test of the tool within the clinic and down the road as we broaden our reach. While the tool is currently being developed with a neuro-oncology caregiving population in mind, it may be flexible enough to be adapted for application in other populations.

### Summative Evaluation

Summative evaluation includes usage and performance criteria. Not only is it important to ensure that people use the technology, it is also important to know that the technology has the desired effect. The expectation of evaluation is another benefit to rolling out a tool in a research setting. Trained researchers are skilled at selecting appropriate, validated measures and participants expect to complete surveys. Thus, it is more likely that higher quality and more complete evaluative feedback is obtained.

In order to prepare for a summative evaluation and ensure that that the design is optimized we are currently conducting a feasibility trial. This trial will include a sample of 40 caregivers of patients with primary brain tumor. In this study, we will collect preliminary data on what we expect to be key outcomes for eSNAP: caregiver social support, burden, and well-being. We will also obtain information about use of social work or counseling services, which we consider an important mechanism for how eSNAP may affect caregivers. In a summative evaluation we may expect our tool to change users’ support, either through heightened awareness of availability or through recommendations to meet with social workers who have access to caregiver social network visualizations. We hypothesize that more at-risk caregivers will meet with social workers and social workers who have easier access to social network information will be better able to tailor recommendations and problem-solve. Based on our conceptual model (Figure 3), we expect increased support to buffer caregiver stress and potentially provide resources to decrease the appraisal of burden, which in turn will improve caregiver quality of life and physical health. However, our main goal at this stage is to obtain feedback on eSNAP’s current design and to determine if a larger trial of the tool is warranted. To do this, we will capture process data, such as how long it takes caregiver participants to use the tool, as well as impressions from clinic staff about the impact of the tool. We will also gather quantitative and qualitative usability and likeability data. Caregiver participants will complete a modified version of a design feedback instrument used in previous research [37-39] and will be asked to provide feedback about what they liked and what they thought could be improved in the tool through open-ended survey questions. This data will be analyzed to inform further refinements of the tool prior to an efficacy trial where we will test the primary psychosocial outcomes identified above.

## Discussion

### Principal Findings

The promise of the Internet as a dissemination tool has interested many researchers in developing Web-based interventions [40]. In addition to challenges that pertain to all intervention development, such as ensuring theoretical underpinnings and selecting an appropriate methodology [41,42], additional challenges exist for Web-based interventions including design considerations and tailoring content to a broader, more diverse audience [40]. Using the CeHRes Roadmap to develop Web-based interventions such as eSNAP helps to address these issues by outlining important milestones and including users and other stakeholders in the process.

There were several key lessons learned in implementing the CeHRes Roadmap that were critical to the development of eSNAP, largely falling under a broader theme of translating theoretical needs and ideas and applying them to tools that need to be effective in the real world. One key lesson was about user values. Although there is well-established literature on caregiver needs, it often is not clear what needs to be addressed simultaneously. For example, while we were able to gather from the literature that engaging existing social support networks would be an important caregiver need, we also learned that caregivers also valued new ways to identify more formal resources, including services within the cancer center and community that can assist families coping with cancer. Caregivers told us that both informal and formal needs were linked together. This led us to add this element to our tool, which ultimately makes it more engaging and useful for caregivers.

A second key lesson was about how design requirements change depending on the modality. Although there is a lot of support for the use of paper-based ecomapping, which creates a visualization in the form of a “web” of support, we found that applying the same information architecture was not intuitive for caregivers when starting from scratch with only the end goal of visualizing a support network in mind. Rather, they preferred to see the data they entered in a list format. Although these processes seem trivial, making the tool “think” the same way as the user facilitates use by reducing frustration. Beyond our specific tool, this has broader implications for translation of theoretical design concepts to practical use [43].

Finally, we learned that it is important to design eHealth programs that support, rather than interfere with systems currently in existence [44]. One major issue with eHealth research is the failure to account for the context. One early decision we had to make was where caregivers would initially access our tool. Initially, we had hoped to leverage the Web-based tool to allow users to access it from anywhere. However, providers in their focus group worried about caregivers in distress not having a safety net and recommended that the initial use happen with easy clinical access, though because the tool is Web-based, later interactions may happen at home. Thus, we revised our plan to integrate eSNAP with the existing social work system to streamline existing services provided, rather than circumventing or replacing them.

By explicitly calling for evaluation, the CeHRes Roadmap also provides important insight into next steps. Although our current goals for eSNAP are to establish feasibility, our ultimate goal is to create an efficacious tool that can be implemented into clinical practice. To do this, we can create outcome benchmarks to establish success; if those are not met, we can return to different points within the CeHRes process to make adjustments. For example, we can return to caregivers to investigate ways to improve the design and functionality of eSNAP or we can return to providers to investigate better ways to integrate into clinical practice. This also hints at how the tool could be adapted for other populations. Further research could investigate how different types of caregivers use eSNAP and how the tool impacts their experience in obtaining social support.

The primary immediate outcome targeted by eSNAP is caregiver social support, which we believe will buffer objective stressors of caregiving to improve caregiver quality of life and physical health. Successfully improving these outcomes in caregivers can have important implications for how clinical care is delivered and for caregiver health. Within the current healthcare system, shortcomings exist with respect to targeted and tailored referrals and delivery of psychosocial support services.[45] In addition, there is a call for tools to assist oncologists in providing family-centered psychological care services to ensure high quality cancer care [46]. Our work and others’ have shown that high support resources for caregivers, especially early in the cancer care trajectory, decreases the burden of care and caregiver stress [20,21,31,47-49]. Lack of support and stress in caregiving has been linked to physical health outcomes including future heart disease and chronic pain, and psychological health outcomes including depression in caregivers [50,51]. Protecting cancer caregivers not only improves the cancer treatment experience and allows for better patient care, but has implications for future health years later. Well-developed Web-based interventions, such as eSNAP, can play an important role in providing high quality, family-centered care.

### Limitations

In some of our early-stage pilot work, our participant samples were relatively homogeneous and small. However, at these stages our goal was directed more towards verifying the conclusions we had drawn from the scientific literature and getting input and insight about more “real world” issues and values. Similarly, some of the data that we received from users is of a qualitative nature and the instruments used to obtain data are not broadly validated. This limits the generalizability of the results and limits the conclusions that can be drawn. However, because we were only validating design decisions prior to conducting a formal summative evaluation, the data is useful to inform our designs. As we progress through the CeHRes stages and our goals change, our studies include more participants, increase diversity and outcomes are measured more rigorously. Obtaining broader input may impact our sense of the user needs and tool requirements or specifications. However, the CeHRes Roadmap is inherently recursive as Web-based intervention development needs to be an iterative process. The Roadmap provides a framework for revisiting these stages as new information emerges or evolves.

### Conclusion

As more eHealth interventions are introduced, the implications of their design and development for clinical practice become more pronounced. Those tools that are developed in frameworks such as the CeHRes Roadmap, which encourages the involvement of end-users in the development process, will be more suited for use in their intended populations, be better tailored for implementation in the intended environment, and will be better able to show evidence for their efficacy. Well-developed tools will make important contributions to improve patient and family health. If patients, families, and clinicians have good experiences with these tools, they may be more likely to use or recommend eHealth interventions in the future. By addressing how eSNAP is meant to be used effectively in real-world settings and establishing benchmarks for success through the CeHRes Roadmap, we will be in a better position to ensure that it will be effective and remain in use, helping families in the long-term.

## Abbreviations

CeHRes: Center for eHealth and Wellbeing Research
eHealth: electronic health
eSNAP: electronic Social Network Assessment Program
